# Integrated Metabolome and Transcriptome Analysis of Petal Anthocyanin Accumulation Mechanism in *Gloriosa superba* ‘Rothschildiana’ during Different Flower Development Stages

**DOI:** 10.3390/ijms242015034

**Published:** 2023-10-10

**Authors:** Yue Sun, Pinli Hu, Yanan Jiang, Jun Li, Jiaxing Chang, Huihui Zhang, Haojing Shao, Yiwei Zhou

**Affiliations:** 1 Shenzhen Branch, Guangdong Laboratory of Lingnan Modern Agriculture, Genome Analysis Laboratory of the Ministry of Agriculture and Rural Affairs, Agricultural Genomics Institute at Shenzhen, Chinese Academy of Agricultural Sciences, Shenzhen 518000, China; sunyue@caas.cn (Y.S.); hupinli@caas.cn (P.H.); jiangyanan@caas.cn (Y.J.); lijun@caas.cn (J.L.); changjiaxing@caas.cn (J.C.); 19sunny@live.cn (H.Z.); 2 Guangdong Key Lab of Ornamental Plant Germplasm Innovation and Utilization, Environmental Horticulture Research Institute, Guangdong Academy of Agricultural Sciences, Guangzhou 510640, China

**Keywords:** *Gloriosa superba*, flower color, anthocyanins, metabolome, transcriptome

## Abstract

Flower color is a key ornamental trait in plants. The petals of *Gloriosa superba* ‘Rothschildiana’ petals undergo a color transformation from yellow to red during their development, but the molecular mechanism of this process remains unexplored. This study examines the anthocyanin profiles and gene expression patterns of ‘Rothschildiana’ petals across four developmental stages: bud (S1), initial opening (S2), half opening (S3), and full opening stage (S4). A total of 59 anthocyanins were identified with significant increases in cyanidin-3,5-*O*-diglucoside, cyanidin-3-*O*-glucoside, pelargonidin-3-*O*-glucoside, and pelargonidin-3,5-*O*-diglucoside levels observed during petal maturation. Transcriptome analysis revealed 46 differentially expressed genes implicated in flavonoid and anthocyanin biosynthesis. Additionally, three gene modules were found to be associated with anthocyanin accumulation throughout flower development. Expression levels of genes associated with auxin, abscisic acid, brassinosteroid signaling, and transcription factors such as NACs and WRKYs underwent significant changes and exhibited strong correlations with several flavonoid and anthocyanin biosynthetic genes in these modules. These findings offer novel insights into the molecular underpinnings of flower color variation and lay the groundwork for the improvement of *G. superba*.

## 1. Introduction

Flower color, a significant ornamental trait of ornamental plants, is influenced by various factors, including pigment substances, cell morphology, and vacuolar pH. Among these, pigment substances such as chlorophyll, carotenoids, and flavonoids are the main causes of large color differences [1]. Anthocyanins, a type of flavonoid compound, can attract animals for seed dispersal and pollination and protect plants from excessive light damage [2,3]. Moreover, anthocyanins have positive effects on human health, exhibiting anti-oxidation, anti-inflammation, and anti-obesity as well as aiding in the prevention of cardiovascular diseases. Therefore, they can serve as potential medicinal ingredients [4,5,6].

Anthocyanins are water-soluble secondary metabolites in plants that belong to six groups: pelargonidin, cyanidin, peonidin, delphinidin, petunidin, and malvidin [7]. Statistical data indicate more than 500 different anthocyanins have been isolated from plants. The biosynthesis pathway of anthocyanins is relatively conserved. It starts with chalcone synthase (CHS) and chalcone isomerase (CHI), which convert one molecule of *p*-coumaroyl-CoA and three molecules of malonyl-CoA into naringenin chalcone. This is then converted into naringenin. Flavonone 3-hydroxylase (F3H) catalyzes the central ring oxidation to produce dihydrokaempferol (DHK), which can be further catalyzed by flavonoid 3′-hydroxylase (F3′H) and flavonoid 3′5′-hydroxylase (F3′5′H) to produce dihydroquercetin (DHQ) and dihydromyricetin (DHM), respectively. Dihydroflavonols (DHK, DHQ, and DHM) can be further converted into pelargonidin-, cyanidin-, and delphinidin-derived anthocyanins by dihydroflavonol reductase (DFR), leucoanthocyanidin dioxygenase (LDOX, also known as ANS or anthocyanidin synthase), and 3-glucosyltransferase (3GT), respectively [8,9,10].

Certain transcription factors (TFs) modulate the biosynthesis and accumulation of anthocyanins by binding to the promoter regions of anthocyanin synthesis-related structural genes. For example, in *Arabidopsis thaliana*, anthocyanin biosynthesis can be controlled by the TTG1–bHLH–MYB regulatory complex [11]. In addition, CPC (CAPRICE) competes with the activator R2R3-MYB TFs PAP1/2 to negatively regulate anthocyanin accumulation [11,12]. In lily flowers, two R2R3-MYB TFs, LvMYB5 and LvMYB1, were found to regulate anthocyanin biosynthesis [13]. AaMYB3 and AabHLH1 are involved in the regulation of proanthocyanidins biosynthesis in anthurium and could potentially be used to metabolically engineer PA biosynthesis in plants [14]. In addition to the MBW complex, some other TFs can also regulate anthocyanin biosynthesis. In apple, MdWRKY11 regulates anthocyanin accumulation in red-fleshed apple by affecting MYB TFs and light-responsive factor MdHY5 [15]. MdERF38 promotes drought stress-induced anthocyanin biosynthesis in apple [16]. In the regulation of anthocyanin biosynthesis in citrus, *CsRuby1* and *CsMYB3* constitute a regulatory loop that functions as an ‘Activator-and-Repressor’ [17]. 

Hormonal signaling pathways orchestrate interactions at the gene expression level to regulate various physiological responses and developmental processes of plants, such as defense responses, plant growth, and anthocyanin accumulation [18,19,20]. For instance, cytokinin enhances anthocyanin content and the expression of the sugar-inducible structural gene UDPglucose: *flavonoid 3-O-glucosyl transferase* (*UF3GT*) and the regulatory gene *production of anthocyanin pigment 1* (*PAP1*) in *Arabidopsis* seedlings [21]. Jasmonate-induced anthocyanin accumulation in *Arabidopsis* requires the COI1 and DFR genes [22]. Ethylene-induced color bleaching in petals of cut *Vanda* ‘Sansai Blue’ flowers results from in-planta anthocyanin degradation, which is partly mediated by increased peroxidase activity and is independent of flower senescence [23].

*Gloriosa superba* L., commonly known as flame lily or glory lily, is a flowering plant in the family Colchicaceae. It has environmental and social uses as well as being a popular ornamental plant. It is the national flower of Zimbabwe, where it is protected. *G. superba* ‘Rothschildiana’ is one of the main varieties cultivated today. Its petal color changes dramatically during development, from yellow-green to dark purple, then to orange-red and finally to bright red. However, the pigments and genes responsible for these color changes are still poorly understood, limiting the quality maintenance and genetic improvement of *Gloriosa superba*. In this project, we used precise colorimeter, ultraviolet spectrophotometer, high-performance liquid chromatography tandem mass spectrometry (HPLC-MS/MS) and Illumina sequencing methods to analyze the differences of anthocyanins and gene expression in petals of four different stages of *G. superba* ‘Rothschildiana’, providing new insights into the color change process of *G. superba* flower in different development stages.

## 2. Results

### 2.1. Differences of Color and Total Anthocyanin in G. superba ‘Rothschildiana’ Petals at Different Flower Stages

**Figure 1:**
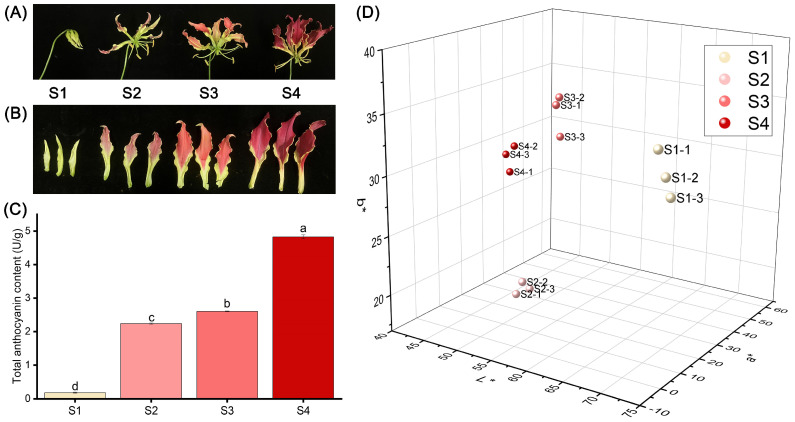
Differences in appearance, anthocyanin content and phenotype of ‘Rothschildiana’ petals during flower development. (**A**,**B**) Changes in the appearance. (**C**) The total anthocyanin content. (**D**) Comparison of L*, a*, and b* values. Differences were defined using least significant difference (LSD) at *p* < 0.05. Significantly different groups were indicated by different letters (a, b, c, d) at *p* < 0.05.

The flower color of ‘Rothschildiana’ undergoes a significant transformation from the bud stage to the full flowering stage (Figure 1A,B). The petals are yellow-green at stage S1, red at stage S2, and then deepen to purple-red at stage S4. As depicted in Figure 1C, the total anthocyanin content in the petals increased gradually from stage S1 to S4, ranging from 0.18 to 4.83 U/g (Figure 1C), with significant differences (*p* < 0.05) among the stages. The total content at stage S4 was 26.92 times that of stage S1. The L*a*b* three-dimensional coordinates showed that the L*, a*, and b* values of the four stages were significantly different. L* decreased with flower development, a* increased with flower development, and b* was the highest at stage S1, the lowest at stage S2, slightly increased at stage S3, and slightly decreased at stage S4.

### 2.2. Metabolomic Profiling

**Figure 2:**
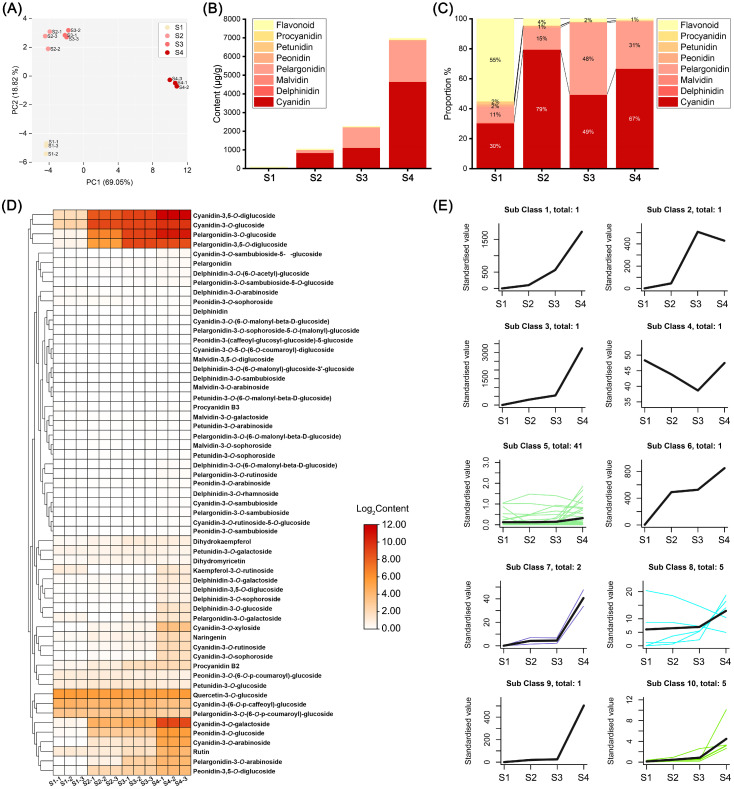
Anthocyanin metabolomic analysis of ‘Rothschildiana’ flower petals at different developmental stages. (**A**) PCA score plots for all samples. (**B**) Total content of various pigment types at four stages. (**C**) The percentage of different pigment types at four stages. (**D**) Heat map of anthocyanin-related metabolite expression. (**E**) Distinct expression patterns of anthocyanin-related metabolites. Different color lines represent the accumulation patterns of various metabolites at different stages.

To elucidate the effects of anthocyanin-related metabolites on flower color change, we analyzed the anthocyanins of ‘Rothschildiana’ flower petals at four different developmental stages using high-performance liquid chromatography (HPLC) and tandem mass spectrometry (MS/MS). By cross-referencing the mass spectrometry data with compound information in the database, we identified 59 anthocyanin-related metabolites, including 13 cyanidins, 11 delphinidins, 4 malvidins, 11 pelargonidins, 7 peonidins, 5 petunidins, 2 procyanidins and 6 flavonoids. To understand the overall characteristics of the metabolite dataset, we first performed principal component analysis (PCA) (Figure 2A). PC1 and PC2 explained 69.05% and 18.82% of the metabolite distribution in the samples, respectively. The four sample groups were distinctly separated. The distance between S2 and S3 groups was relatively close, indicating that they had similar anthocyanin contents, while S1 and S4 groups were far away from the previous two groups, indicating significant differences in anthocyanin contents at different flower developmental stages. The total content analysis (Figure 2B,C) revealed that the total anthocyanin content in the petals increased with flower development. The total contents at stages S1 to S4 were 92.13, 1054.49, 2262.82 and 6975.60 μg/g respectively, which was consistent with the flower color phenotype. Flavonoids constituted the main components at stage S1, accounting for 55% of the total content. At stages S2–S4, cyanidin and pelargonidin pigments accounted for more than 94% of the total content. Therefore, cyanidin and pelargonidin pigments were the main pigment components of ‘Rothschildiana’ after stage S2.

Hierarchical clustering heat map analysis of 59 anthocyanin metabolites found that some major pigment substances with relatively high contents accumulated with the change in stages (Figure 2D), including cyanidin-3,5-*O*-diglucoside, cyanidin-3-*O*-glucoside, pelargonidin-3-*O*-glucoside, and pelargonidin-3,5-*O*-diglucoside, which clustered into a small group. K-means clustering analysis partitioned the contents of 59 anthocyanins at different flower developmental stages, which could be divided into ten clusters (Figure 2E), among which eight clusters containing fifteen anthocyanins had expression patterns that increased with stage change, including cluster 1, 2, 3, 6, 7, 8, 9 and 10 (Table S1). Among them, five anthocyanins in cluster 8 only had three kinds that gradually increased with flower development. This suggests that most of the anthocyanins in *G. superba* petals accumulated with flower development, which was consistent with the flower color phenotype change pattern.

### 2.3. Transcriptome Analysis

**Figure 3:**
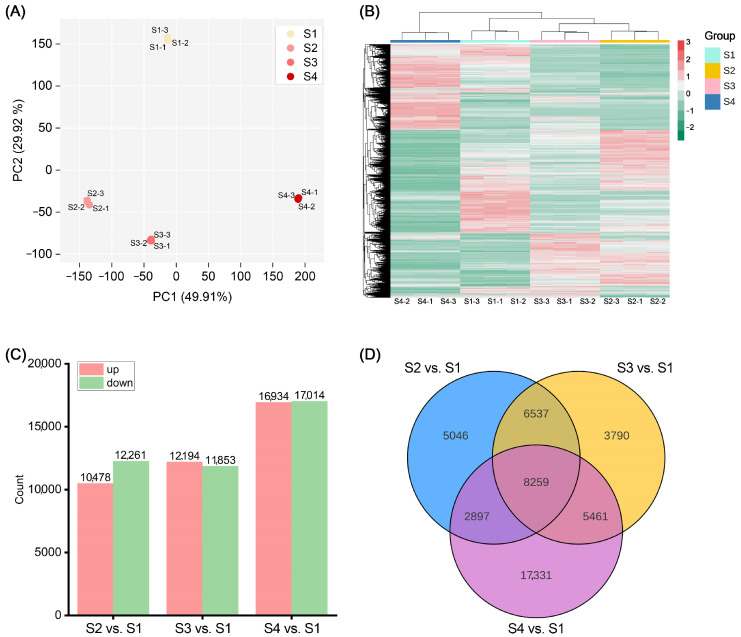
Analysis of transcriptome and differentially expressed genes. (**A**) PCA score plot of 12 transcriptome samples. (**B**) Heat map of all genes’ relative expression with Log_2_FPKM. (**C**) Number of differentially expressed genes (DEGs) among S2 vs. S1, S3 vs. S1, and S4 vs. S1. (**D**) Venn plot of DEGs among S2 vs. S1, S3 vs. S1, and S4 vs. S1.

To identify key genes affecting anthocyanin accumulation in ‘Rothschildiana’ flower petals, we performed RNA-seq analysis on petals from four different developmental stages of ‘Rothschildiana’. PCA and clustering heat map analysis showed that there was little difference within each group of samples, indicating good data reproducibility, while there was a significant difference between groups (Figure 3A,B). RNA-seq data yielded in 9.49–11.26 GB clean bases with an error ratio of less than 0.05%, a Q20 value higher than 96.2%, a Q30 value greater than 90.8%, and a GC percentage ranging from 48.30 to 49.42% (Table S2). Table S3 presents the length and number of assembled transcripts and genes. Gene annotation results indicate that the KEGG, NR, Swiss-Prot, GO, KOG, Trembl, and Pfam databases annotated 58,154, 76,316, 57,961, 76,401, 46,117, 66,047 and 53,385 genes, respectively, for a total of 101,525 annotated genes (Table S4). Among them, 38,394 genes were commonly annotated by KEGG, NR, Swiss-Prot, GO and Pfam (Figure S4). Differential gene expression analysis revealed that there were 10,478 upregulated genes and 12,261 downregulated genes in the S2 vs. S1 comparison (Figure S1); 12,194 upregulated genes and 11,853 downregulated genes in the S3 vs. S1 comparison (Figure S2); and 16,934 upregulated genes and 17,014 downregulated genes in the S4 vs. S1 comparison (Figure 3C; Figure S3). Venn diagram analysis showed that there were 8259 differentially expressed genes common to the S2 vs. S1, S3 vs. S1, and S4 vs. S1 comparisons (Figure 3D). This suggests that the expression of many genes changed significantly with petal development.

**Figure 4:**
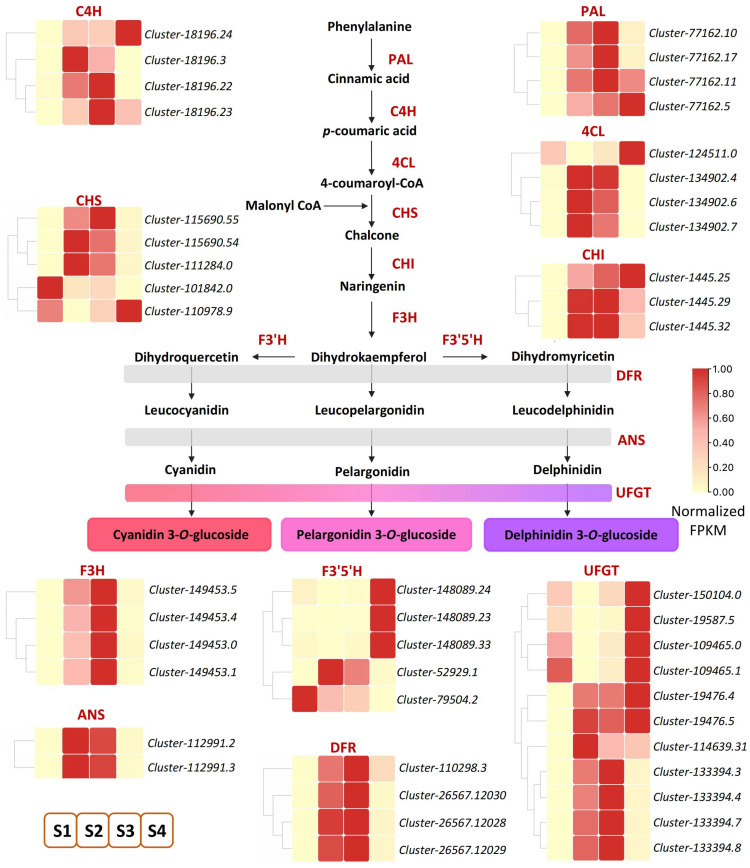
DEGs of anthocyanin biosynthesis pathway in ‘Rothschildiana’ petals.

Metabolome data analysis showed that anthocyanin-related metabolites increased significantly. To further identify key genes involved in anthocyanin accumulation in ‘Rothschildiana’ petals, we pinpointed 46 DEGs related to anthocyanin biosynthesis among the differentially expressed genes (Figure 4; Table S5). Anthocyanins belong to flavonoids, which share a common synthesis main chain with other flavonoids upstream and form various anthocyanins by branch synthesis reactions from dihydrokaempferol. As depicted in Figure 3, upstream from the elements of the synthesis pathway, most of the *PAL*, *C4H*, *4CL*, *CHI*, and *CHS* genes had higher expression levels at stages S2 and S3, and a few genes had higher expression levels at stage S4, which provided substrates for the synthesis of downstream anthocyanin substances. Downstream of the synthesis pathway, *F3H*, *ANS*, and *DFR* genes, which are involved in the synthesis of colored substances, had the highest expression levels at stages S2 and S3. For the key gene *UFGT* that forms colored substances, most of them had the highest expression level at stage S4, and some had the highest expression level at stages S2 and S3, which was consistent with the total content of flower color substances.

### 2.4. Weighed Gene Co-Expression Network Analysis (WGCNA)

**Figure 5:**
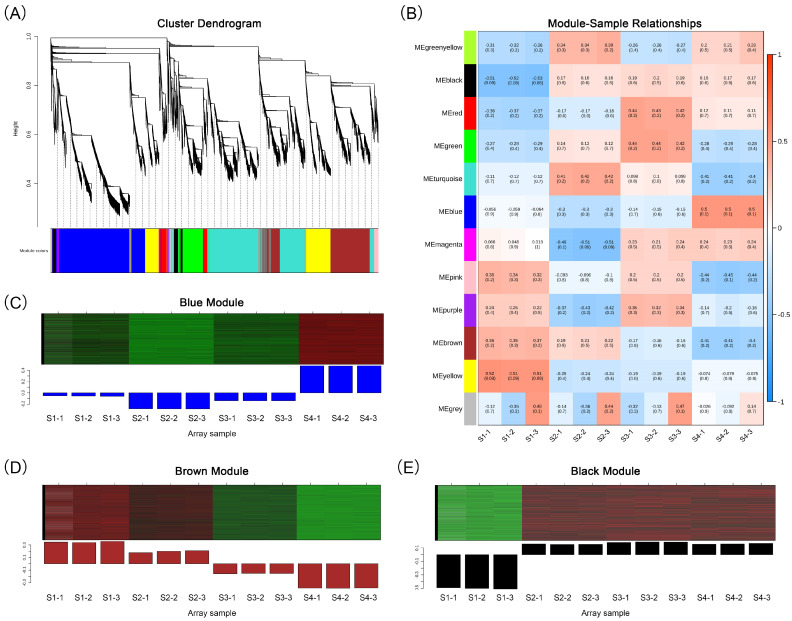
Weighted gene co-expression network analysis. (**A**) Cluster dendrogram of DEGs. (**B**) Correlation heat map between 12 gene modules and 12 samples. (**C**) Relative expression of genes in the blue module. (**D**) Relative expression of genes in the brown module. (**E**) Relative expression of genes in the black module. In the heat map of (**C**–**E**), green color indicates a lower relative expression level of genes, while red color indicates a higher relative expression level of genes.

We conducted WGCNA analysis on 30,818 differentially expressed genes, segregating them into 12 modules (Figure 5A; Table S6). These encompassed the following: black (936 genes), blue (8184 genes), brown (5233 genes), green (2225 genes), green–yellow (62 genes), magenta (249 genes), pink (545 genes), purple (164 genes), red (1026 genes), turquoise (8267 genes), yellow (3781 genes) and gray (146 genes). We established correlations between the modules and the 12 samples as well as 15 major anthocyanins to identify the modules associated with flower development and anthocyanin accumulation. We found that the blue and black modules had significant positive correlations with these traits, while the brown module had a significant negative correlation (Figure 5B and Figure S5). The blue module exhibited the highest expression level at the S4 stage (Figure 5C). The brown module decreased in expression level as the flowers developed and accumulated anthocyanins, while the black module maintained a high expression level in all three stages of flower coloration (Figure 5D). 

### 2.5. Plant Hormone Signaling Pathway Analysis of Important Gene Modules

**Figure 6:**
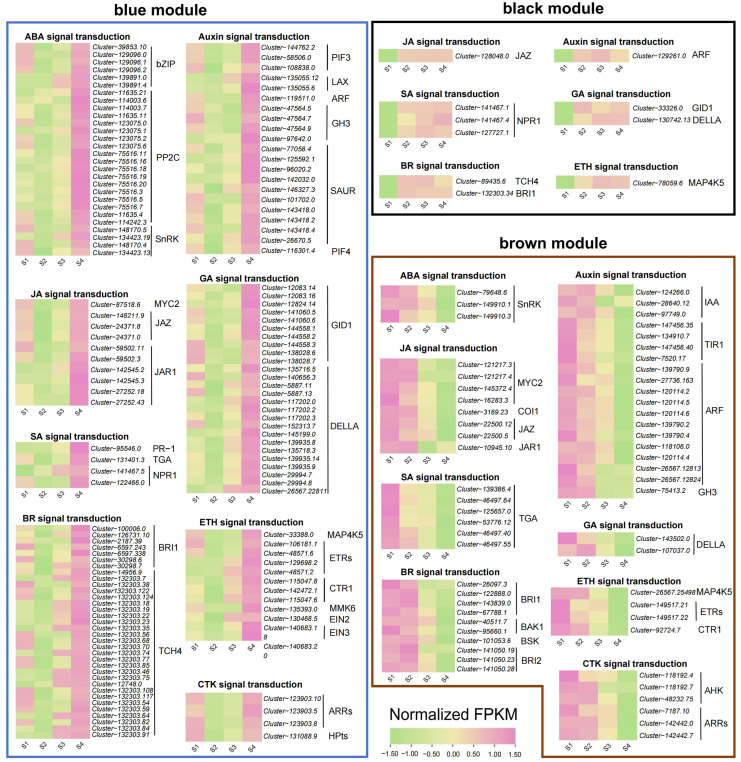
Expression patterns of differentially expressed genes related to plant hormone signal transduction in blue, black, and brown gene modules.

To understand the relationship between the expression of plant hormone-related genes and flower color change, we analyzed the genes annotated as the plant hormone signal transduction (ko04075) pathway in the blue, brown, and black modules of WGCNA results (Table S8, Figure 6). In the blue module, we retained 139 hormone signaling-related genes, most of which had the highest accumulation in the S4 stage, and some also had high expression in the S1 stage. In the brown module, we retained 58 hormone signaling-related genes, most of which were downregulated with the change in stages. In the black module, we retained 11 genes, most of which were upregulated with the change in stages. 

Across the three modules, we determined that all genes were related to eight hormone signaling pathways, namely abscisic acid, auxin, brassinosteroid, jasmonate, gibberellin, ethylene, salicylic acid and cytokinin. Among the genes involved in the abscisic acid signaling pathway, 18 *protein phosphatase 2C* (*PP2C*) genes and six *bZIP* genes reached the highest accumulation in the S4 stage, while *serine/threonine-protein kinase* (*SnRK*) genes showed two opposite expression patterns with the change in stages. Among the genes involved in the auxin signaling pathway, we screened two *auxin influx carriers* (*LAX*) genes that gradually accumulated with the change in stages, while 11 *auxin response factors* (*ARF*) and 4 *transport inhibitor response 1* (*TIR1*) genes were downregulated with the change in stages. In brassinosteroid signaling pathway, we screened 26 xyloglucosyl transferase *TCH4* genes, most of which were upregulated with the change in stages. For the jasmonate signaling pathway, three *MYC2* genes showed a downward trend, and we also mined six *jasmonate ZIM domain-containing* (*JAZ*) genes and six *jasmonic acid-amino synthetase* (*JAR1*) genes that had the highest accumulation in the S4 stage. For the ethylene signaling pathway, we found that one *ethylene-insensitive 2* (*EIN2*) gene and one *ethylene-insensitive 3* (*EIN3*) gene were upregulated with the change in stages.

### 2.6. Correlation Network Analysis

**Figure 7:**
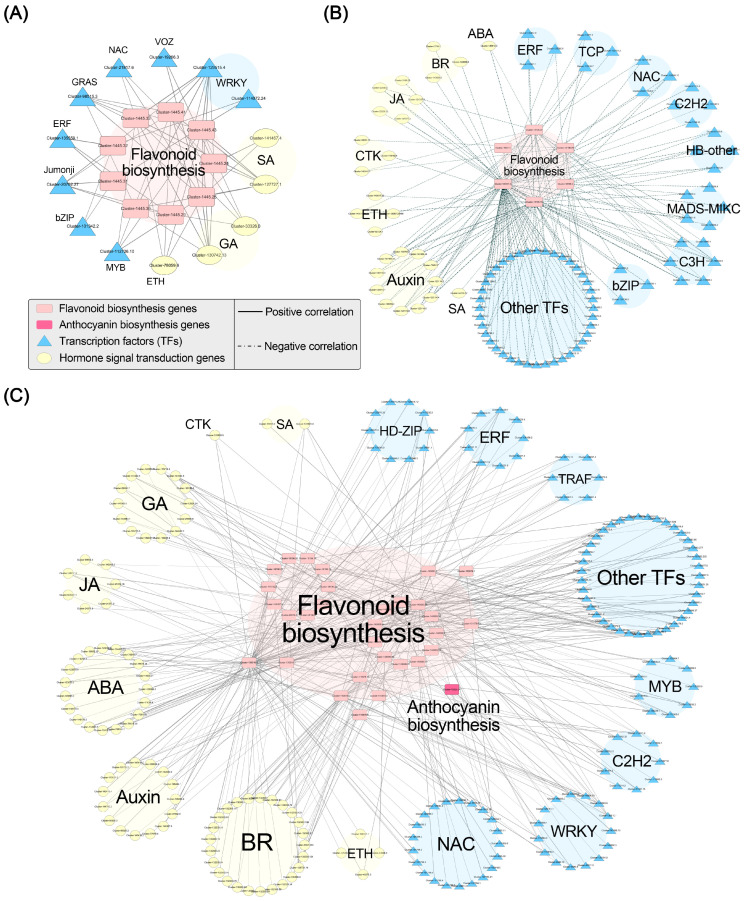
Pearson correlation networks between important flavonoid and anthocyanin biosynthesis genes and transcription factors in black (**A**), brown (**B**), and blue (**C**) gene modules.

We conducted Pearson correlation analysis between flavonoid (including anthocyanin) biosynthesis genes and hormone signaling-related genes in addition to flavonoid (including anthocyanin) biosynthesis genes and TFs. We established correlation networks for the three gene modules with a threshold of *p* < 0.05 and *r* > 0.95 (Figure 7). In the black module, we identified 9 flavonoid biosynthesis genes (Table S8), 11 hormone signaling-related genes (Table S8), and 16 TFs (Table S9). Correlation analysis revealed that nine flavonoid biosynthesis genes (CHI) were significantly positively correlated with 2 SA signaling genes, 2 GA signaling genes, 1 ethylene signaling gene, and 11 TFs. In the brown module, we identified 58 hormone signaling-related genes and 168 TFs that exhibited negative correlations with anthocyanin accumulation (Tables S7 and S9). The correlation analysis with the flavonoid and anthocyanin biosynthesis-related genes in the black and blue modules revealed that six flavonoid biosynthesis-related genes were significantly negatively correlated with 1 ABA signaling gene, 11 auxin signaling-related genes, 3 BR signaling-related genes, 3 CTK signaling-related genes, 4 ethylene signaling-related genes, 5 JA signaling-related genes, 1 SA signaling gene, and 71 TFs. In the blue module, we identified 46 flavonoid biosynthesis genes (Table S8), 2 anthocyanin biosynthesis-related genes, 139 hormone signaling-related genes (Table S8), and 227 TFs (Table S9). The correlation analysis showed that 31 biosynthesis-related genes (30 flavonoid biosynthesis genes and 1 anthocyanin biosynthesis gene) were significantly positively correlated with 19 ABA signaling-related genes, 15 auxin signaling-related genes, 25 BR signaling-related genes, 1 CTK signaling gene, 4 ethylene signaling-related genes, 14 GA signaling-related genes, 7 JA signaling-related genes, 2 SA signaling-related genes, and 114 TFs. These findings suggest that these TFs and hormone signals may participate in the regulation of anthocyanin accumulation in ‘Rothschildiana’ petals by targeting structural gene expression.

### 2.7. Quantitative Real-Time PCR (qRT-PCR) Analysis

**Figure 8:**
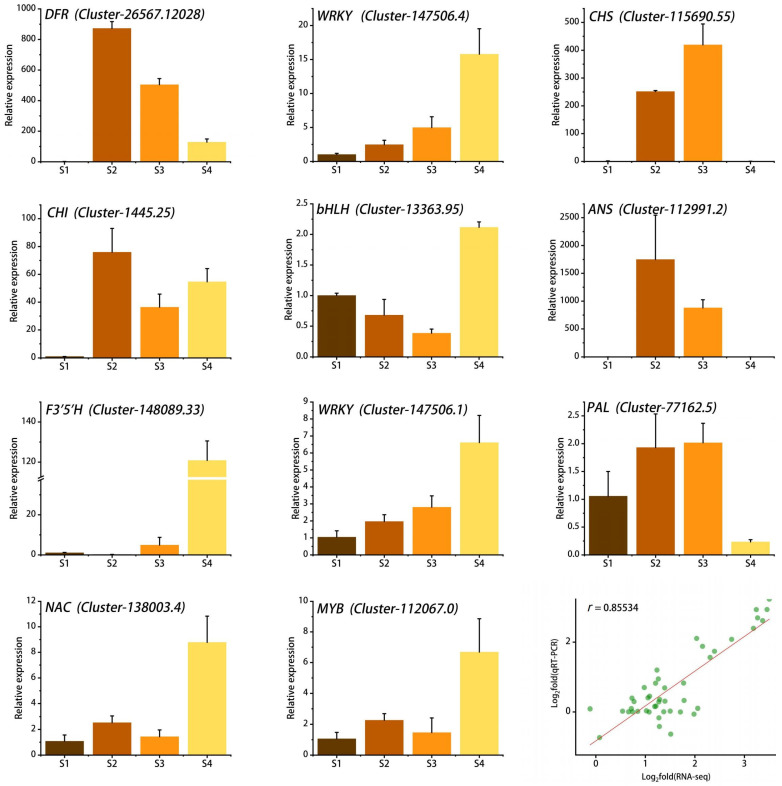
qRT-PCR validation of 11 key genes and correlation analysis with RNA-seq data. Stages S1 ~ S4 are respectively represented by the colors brown, coffee, orange, and yellow. The green dots denote the two-dimensional coordinates of Log2(RNA-seq) and Log2(qRT-PCR), while the red line illustrates the general linear fit of the Log2(RNA-seq) and Log2(qRT-PCR) data.

To verify the accuracy of the RNA-seq data, we randomly selected 11 genes involved in the flavonoid biosynthesis pathway and the WGCNA results for qRT-PCR analysis. The results showed that the expression levels of most genes increased gradually with flower development, which was consistent with the accumulation pattern of anthocyanins in petals. Linear regression analysis showed that the qRT-PCR results were significantly positively correlated with the RNA-seq results, with *r* reaching 0.85, indicating that the RNA-seq data were reliable and accurate (Figure 8).

## 3. Discussion

In the study of plant color, measuring phenotypic color is crucial. While humans can describe color through observation, they are unable to quantify it directly in a scientific manner. An accurate description of flower color phenotype is conducive to classifying varieties by color series and monitoring flower color quality. In their study on gerbera, Zhou et al. [24] utilized a colorimeter to measure and analyze 123 gerbera petals, successfully categorizing them into six color groups. They found that the total carotenoids and total anthocyanins differed significantly among different color series. In caladium, Zhou et al. [25] used a colorimeter combined with cluster analysis to divide 154 caladiums’ leaf color into four groups. In chrysanthemum, Lu et al. [26] used a colorimeter combined with cluster analysis to successfully divide 273 pot multi-flowered chrysanthemum varieties into six color groups. They analyzed anthocyanins and carotenoids by HPLC/MS and found that the a* value was significantly correlated with anthocyanins and the b* value was significantly correlated with carotenoids. In this study, we also used a colorimeter to analyze the color of ‘Rothschildiana’ petals at four different developmental stages. We observed that the colorimetric detection values of petal color at four stages exhibited significant differences, and the a* value increased continuously with flower development, aligning with the change pattern of total anthocyanin content. These studies all indicate that colorimetric detection is an efficient method for analyzing flower color phenotype.

Among omics tools, metabolomics emerges as a fascinating tool widely employed in crop improvement [27]. Metabolomics complements other omics approaches as it represents the “downstream” result of gene expression Changes in metabolomics are considered to best mirror cellular activity at the functional level [28]. Therefore, it can be used to detect changes in plant metabolites under different background factors and then carry out targeted improvement and related evaluation. In this study, we extracted anthocyanins from petals of ‘Rothschildiana’ at different developmental stages and performed metabolomic detection. The results showed that 62 substances were detected in four groups of samples, such as cyanidin-3,5-*O*-diglucoside, cyanidin-3-*O*-glucoside, pelargonidin-3-*O*-glucoside, pelargonidin-3,5-*O*-diglucoside, etc., which accumulated continuously with flower development. The percentage of anthocyanins in total metabolites increased, resulting in deepening petal color. At the same time, we found that cyanidin and pelargonidin derivatives were the main substances in S2–S4 periods when red appeared, while flavonoid was the main substance in the S1 period when yellow appeared. Interestingly, the percentage of cyanidin and pelargonidin classes changed dynamically in the S2–S4 periods. The percentages of cyanidin and pelargonidin classes in total material content were 79% and 15%, 49% and 48%, and 67% and 31% in the S2–S4 periods, respectively (Figure 2C). This dynamic percentage affects petal color to some extent. The petals of the S2 period were dark purple, while those of the S3 period were red–orange, and those of the S4 period were purple–red. According to the literature reports, cyanidin, pelargonidin and delphinidin can, respectively, present red to purple, orange, and purple to blue [23,24,25]. Therefore, during the S3 period, the ‘Rothschildiana’ cyanidin and pelargonidin classes were about 1:1 and the petal color was red–orange, while during the S1 and S4 periods, the cyanidin content percentage was higher than pelargonidin, and the petal color was red–purple.

The chemical structure of anthocyanin aglycone is inherently unstable and can be readily altered within plant cells. In order to form more stable compounds, plants promptly synthesize anthocyanin aglycone after chemical reactions such as acylation, glycosylation, and methylation [10,29]. Within the anthocyanin biosynthesis pathway, a series of genes participate, including early pathway genes (*PAL*, *C4H*, *4CL*, *CHS*, *CHI*, and *F3H*) and late pathway genes (*DFR*, *ANS*, and *UFGT*) [30,31,32]. The UGFT enzyme is the final gene in the anthocyanin pathway and is key to stabilizing anthocyanin [33,34]. We identified 11 *UFGT* genes from the transcriptome differential genes. Four genes (*Cluster-133394.3*, *Cluster-133394.4*, *Cluster-133394.7*, *Cluster-133394.8*) were highly expressed only in S2 and S3 periods, with higher expression levels in S3 than S2. Six (*Cluster-150104.0*, *Cluster-19587.5*, *Cluster-109465.0*, *Cluster-109465.1*, *Cluster-19476.4*, and *Cluster-19476.5*) were most highly expressed in the S4 period. These genes may be important for the stable coloring and deepening of cattleya petal color. In morning glory, the absence of UFGT results in a pale flower color [35]. The gene silencing of *PeUFGT3* in *Phalaenopsis* leads to a decrease in anthocyanin content, indicating that the glycosylation-related gene *PeUFGT3* plays a key role in the formation of red color in *Phalaenopsis* [36]. Similarly, in Japanese apricot petals, the UFGT enzyme activity of red petals was significantly higher than that of white petals. When red spots appeared on white petals, the enzyme activity increased synchronously with anthocyanin accumulation [37].

Transcription factors such as MYB, bHLH, WD40, and MADS-box also regulate anthocyanin biosynthesis [38]. In *Dendrobium* hybrid petals, transient overexpression showed that *DhMYB2* and *DhbHLH1* both led to anthocyanin production in white petals [39]. Homozygous mutants (*OsTTG1*) produced by CRISPR/Cas9 showed a significant reduction in anthocyanin accumulation in various organs of rice. The WD40 gene *OsTTG1* is considered an important regulatory factor for anthocyanin biosynthesis in rice [40]. Virus-induced gene-silencing (VIGS) experiments on *Senecio cruentus* showed that silencing two *MADS-box* genes (*ScAG* and *ScAGL11*) increased the anthocyanin content in *Senecio cruentus* leaves [41]. Compared with the control, the overexpression of *PpNAC25* in poplar resulted in redder stem tips and could bind to and activate transcripts of the promoters of anthocyanin activators *PpMYB10.1* and *PpMYB10.2*. This indicates that PpNAC25 may positively regulate peach fruit anthocyanin biosynthesis [42]. In this study, we identified transcription factors related to anthocyanin accumulation during the flower development of ‘Rothschildiana’ by analyzing WGCNA combined with correlation network analysis. These include NAC, WRKY and MYB and other TFs family genes which were significantly positively correlated with multiple flavonoid biosynthesis-related genes and may have important functions in regulating anthocyanin synthesis in ‘Rothschildiana’ petals. These transcription factors require further experimental verification.

Plant hormones participate in every stage of plant development. In this study, we discovered that the expression of genes in eight endogenous hormone-signaling pathways exhibited significant differences, indicating that *Gloriosa superba* petals were regulated by endogenous hormones during development. The treatment of sweet cherry fruit with ABA elevated the transcript abundance of anthocyanin pathway-related genes *PavF3H* and *PavCHS* along with the ABA signaling pathway-related genes *PavPP2C3*, *PavPP2C4* and *PavSnRK2.1* [43]. In *Gloriosa superba*, we also identified 18 *PP2C* and four *SnRK2* genes that accumulated concurrently with anthocyanin accumulation. In apple (*Malus* × *domestica*), *MdARF2* was identified as a transcriptional repressor of anthocyanin biosynthesis [44]. These were similar to the 11 ARFs in the brown module of *Gloriosa superba*, which decreased in expression with flower development. Methyl jasmonate (JA-Me) at concentrations of 0.1, 0.5 and 1.0% (*w*/*w*) greatly stimulated the anthocyanin accumulation in shoots of young plants of *Kalanchoe blossfeldiana* when it was applied around the stem as a lanolin paste [45]. The FtJAZ2 interacted with the R2R3-MYB transcription factor FtMYB3 to affect anthocyanin biosynthesis in tartary buckwheat [46]. In *Arabidopsis* seedlings, JA-induced anthocyanin accumulation was reduced in BR mutants or in wild type treated with brassinazole, an inhibitor of BR biosynthesis, whereas it was induced by an application of exogenous BR [47]. Similarly, in *Gloriosa superba* flower development, jasmonate and brassinosteroid signaling pathway-related genes were generally upregulated and reached the highest level during the S4 stage. How these plant hormone signals interact with which proteins to regulate the expression of anthocyanin biosynthesis-related pathway genes and thus regulate anthocyanin accumulation in *Gloriosa superba* requires further in-depth study.

## 4. Materials and Methods

### 4.1. Plant Materials

*G. Superba* ‘Rothschildiana’ were planted in the Dingdong Fragrant Garden (23°27′ N, 113°15′ E), Foshan, Guangdong, China. During our observation of the growth and development of ‘Rothschildiana’, we noticed a color change phenomenon from the initial flowering stage to the full flowering stage. We divided this period into four stages for further study: bud stage (S1), initial opening stage (S2), half opening stage (S3), and full opening stage (S4). Fresh petals were collected, immediately placed in liquid nitrogen, and stored at −80 °C for RNA extraction and anthocyanin extraction.

### 4.2. Phenotypic Determination of Petal Color

Following the method of Zhou et al. [25], we determined the main color of the petals using a 3nh NR110 colorimeter (3nh Technology Co., Ltd., Shenzhen, China) and employed the parameters of the CIELab* color coordinate (lightness, L*; chromatic components, a* and b*). Each sample was measured five times.

### 4.3. Total Anthocyanin Content Detection

The method for measuring total anthocyanin (At) content was adapted from [48]. Each sample, weighing 300 mg, was ground into a fine powder using liquid nitrogen. The powder was then transferred to a pre-prepared 5 mL extraction solution of HCl and methanol (1:99 *v*/*v*) and stored at 4 °C for 24 h. After centrifuging the solution at 13,000 rpm for 20 min, the supernatant was transferred to a new centrifuge tube. The optical density value (OD) of the solution at A530 was measured using a UV-1200 spectrophotometer (MAPADA, Shanghai, China). The formula for calculating the total anthocyanin (At) content was QAt = A530 × M^−1^, where QAt is the amount of At and M is the fresh weight (g) of the plant material used for extraction. Each sample had three replicates.

### 4.4. Liquid Chromatography and Mass Spectrometry

Sample preparation, extract analysis, metabolite identification, and quantification were performed at Wuhan Metware Biotechnology Co., Ltd. (Wuhan, China). Chromatographic mass spectrometry data were collected using ultra-high performance liquid chromatography (UPLC, ExionLC™ AD) and tandem mass spectrometry (MS/MS, QTRAP^®^, 6500+) with an ACQUITY BEH C18 1.7 µm × 2.1 mm × 100 mm chromatographic column. Ultra-pure water (with 0.1% formic acid added) was used as mobile phase A and methanol (supplemented with 0.1% formic acid) was used as mobile phase B. The elution gradient was as follows: at 0 min, the B phase ratio was 5%, increasing to 50% at 6 min, then to 95% at 12 min, maintained for 2 min, decreased to 5% at 14 min, and equilibrated for 2 min with a flow rate of 0.35 mL/min, column temperature of 40 °C, and injection volume of 2 μL. The electrospray ionization (ESI) temperature was set to 550 °C with a mass spectrometry voltage of 5500 V and Curtain Gas at 35 psi. Each ion pair was scanned and detected according to the optimized declustering potential (DP) and collision energy (CE). 

### 4.5. Qualitative and Quantitative Analyses of Metabolites

Quantification was performed by the multiple reaction monitoring mode (MRM) of triple quadrupole mass spectrometry and qualitative analysis of mass spectrometry data based on the MWDB (Metware Database, www.metware.cn, accessed on 14 October 2022) database constructed by anthocyanin standards. We used Analyst software v1.6.3 (Scienx) to collect data and Multiquant software v3.0.3 (Scienx) to quantify all metabolites. To ensure the accuracy of qualitative and quantitative analysis, we corrected the chromatographic peaks of the analytes in different samples based on the retention time and peak shape information of anthocyanin standards in MWDB. We used the following method to identify and measure anthocyanins: We calculated the concentration value (ng/mL) of each sample by substituting the integrated peak area into the standard curve equation (Table S10). We then used the following formula to calculate the levels of metabolites in the sample (μg/g): Levels of metabolites in the sample (μg/g) = c × V/1,000,000/m where c is the concentration value, V is the volume of solution used in extraction (μL), and m is the mass of the weighed sample (g).

### 4.6. RNA-Sequencing (RNA-seq)

RNA-seq analysis was performed on petals collected using the above method with three biological replicates for each period, resulting in a total of 12 samples. The samples were sent to Novogene Bioinformatics Technology Co., Ltd. (Beijing, China) for RNA sequencing. Raw data were filtered and cleaned to obtain high-quality clean reads. For the non-reference transcriptome, clean reads were spliced into Unigene using Trinity [49]. Unigene sequences were compared with KEGG, NR, Swiss-Prot, GO, COG/KOG, Trembl and Pfam databases to obtain annotation information. Unigene expression level was calculated as Fragments Per Kilobase of transcript per Million fragments mapped (FPKM), and DESeq2 [50] was used to screen out differentially expressed genes (DEGs) with screening conditions as |log2Fold Change| ≥ 1 and FDR < 0.05. The transcription factor prediction was performed using the iTAK v1.6 [51] software package.

### 4.7. Quantitative Real-Time PCR (qRT-PCR) Analysis

qRT-PCR analysis was performed on 11 selected DEGs involved in the anthocyanin biosynthesis pathway and important transcription factors. The total RNA was extracted using the RNAprep Pure Plant Plus Kit (TIANGEN, Beijing, China) and then reverse transcribed using Hifair^®^ III 1st Strand cDNA Synthesis SuperMix (YEASEN, Shanghai, China) for fluorescence quantification. The evaluation results of high-quality RNA are presented in Figure S6 and Table S11. Hieff UNICON^®^ Universal Blue qPCR SYBR Green Master Mix (YEASEN, Shanghai, China) was used as the premix. Primers were designed using a web-based tool available at (https://www.ncbi.nlm.nih.gov/tools/primer-blast/, accessed on 27 April 2023) with ACTIN as the internal reference gene. The primers are listed in Table S12. The melting curve is shown in Figure S7. The expression level of genes was calculated by the 2^−ΔΔ*C*T^ method [52].

### 4.8. Statistical Analysis

A one-way analysis of variance (ANOVA) was conducted on the data using SPSS 16.0 (SPSS, Inc., Chicago, IL, USA). The mean values were compared by Duncan’ s multiple range tests at a significance level of *p* < 0.05. All analyses were performed using the R packages unless otherwise stated. Principal component analysis (PCA) was conducted using the “mixOmics” package. Hierarchical clustering heat map analysis was carried out using the “pheatmap” package. K-means clustering was performed using Metware Cloud, which is a free online platform for data analysis. We performed weighted gene co-expression network analysis using the “wgcna” package in R language [53]. We used the built-in function in R language to perform Pearson correlation analysis between important anthocyanins and their biosynthesis-related structural genes, flavonoid or anthocyanin biosynthesis genes and transcription factors. We used Cytoscale 3.9 to visualize the correlation network [54].

## 5. Conclusions

We investigated the anthocyanin metabolites and their associated genes in *G. superba* ‘Rothschildiana’ during flower development. We detected 59 anthocyanin metabolites, primarily Cyanidin and Pelargonidin derivatives, with the total content increasing along developmental stages. We also performed weighted gene co-expression network analysis (WGCNA) and found that genes involved in auxin, abscisic acid, brassinosteroid signaling, and transcription factors such as NACs and WRKYs were co-expressed with several flavonoid and anthocyanin biosynthetic genes in black, blue and brown modules. This elucidated the molecular mechanism of anthocyanin accumulation and synthesis in *G. superba* ‘Rothschildiana’. Our results contribute to the understanding of the biochemical basis of flower color formation in *G. superba*.

## Data Availability

The transcriptome raw data have been submitted to the SRA database of the NCBI (PRJNA1000228).

## Supplementary Materials

The following supporting information can be downloaded at: https://www.mdpi.com/article/10.3390/ijms242015034/s1.
