# Supplementary figures and images for: Integrated Metabolome and Transcriptome Analysis of Petal Anthocyanin Accumulation Mechanism in Gloriosa superba ‘Rothschildiana’ during Different Flower Development Stages
Source: Int J Mol Sci. 2023 Oct 10;24(20):15034. doi: 10.3390/ijms242015034 (PMC10606226; doi:10.3390/ijms242015034)

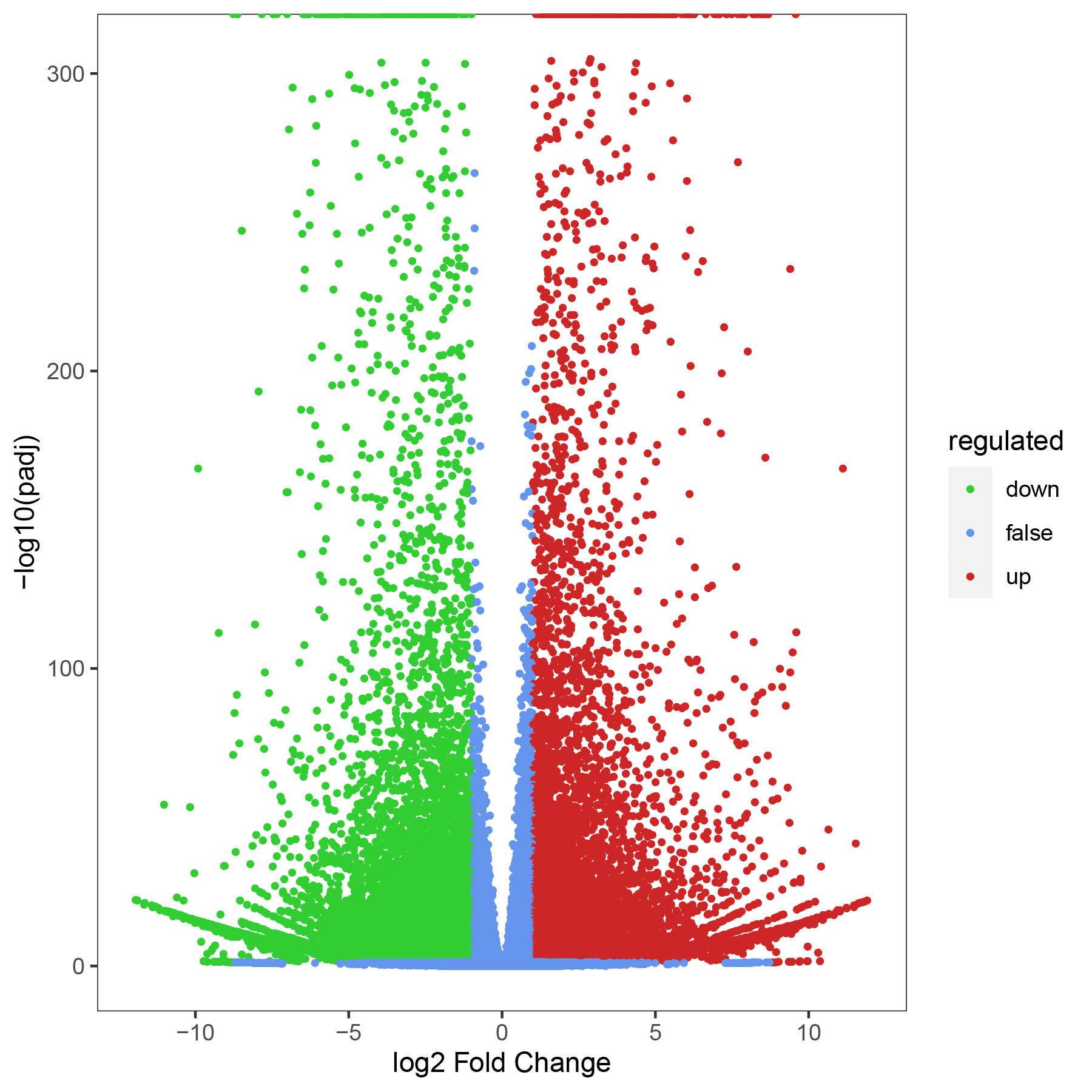

Supplement: Supplementary file 1 [file ijms-24-15034-s001.zip › supplementary materials/Figure S1.jpg]

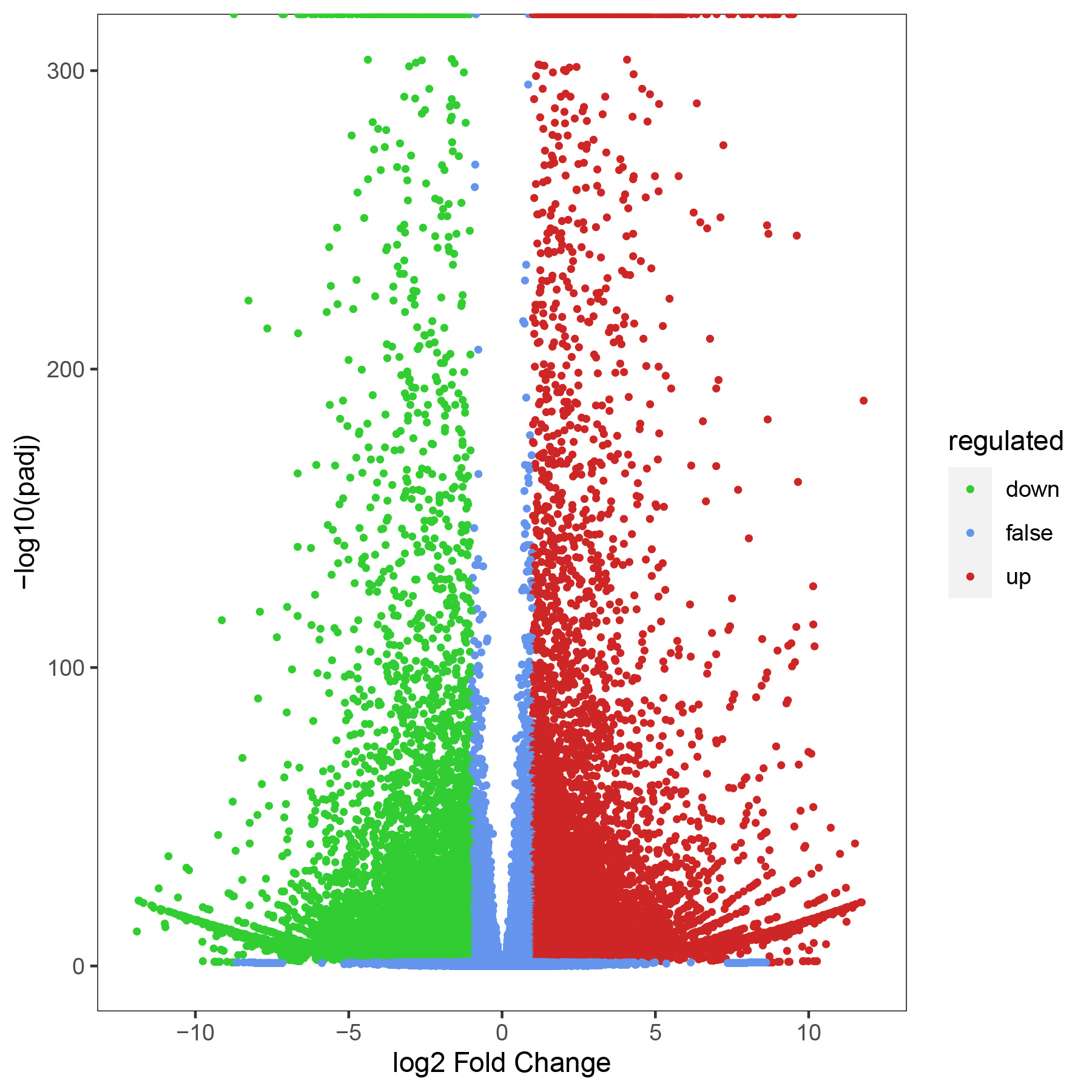

Supplement: Supplementary file 1 [file ijms-24-15034-s001.zip › supplementary materials/Figure S2.jpg]

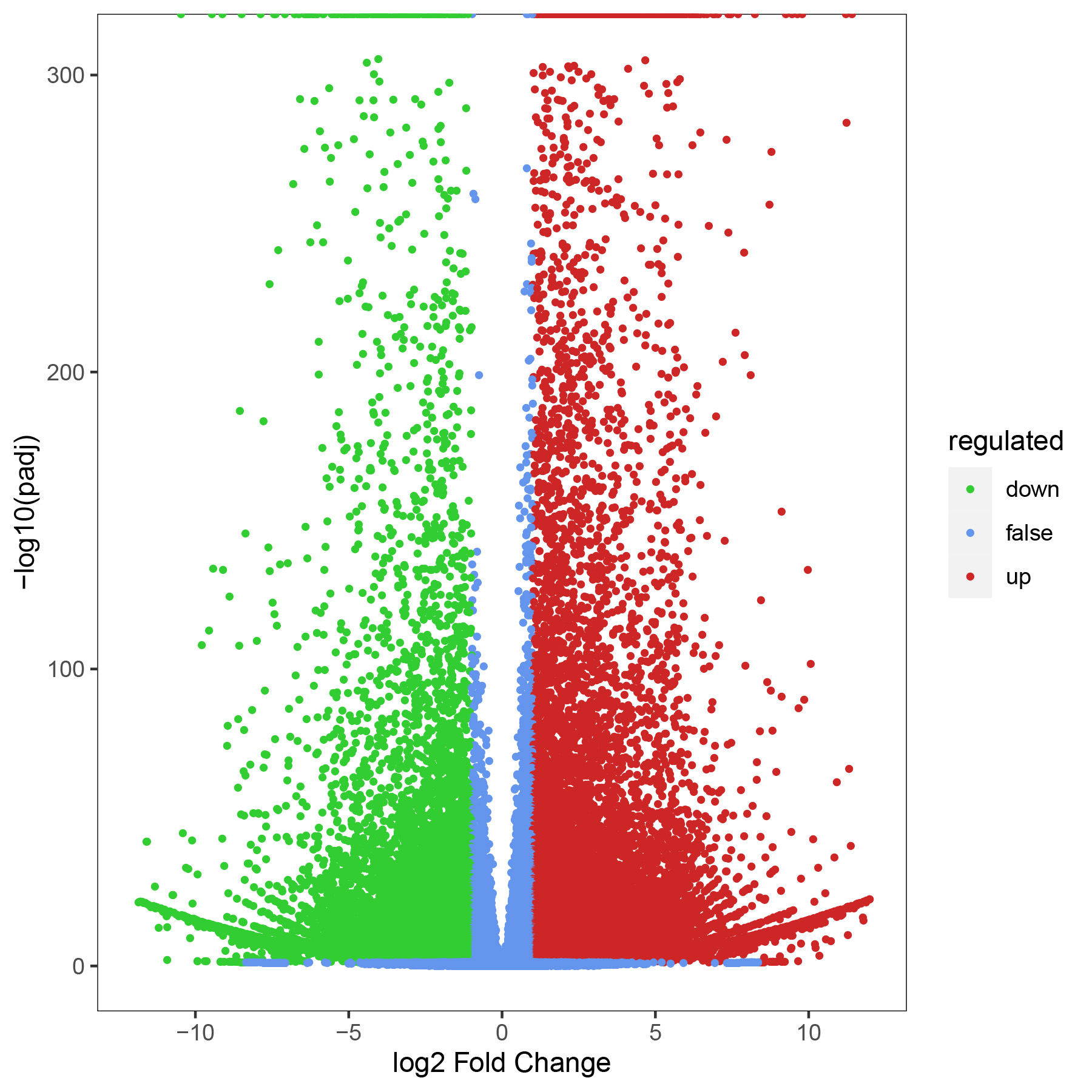

Supplement: Supplementary file 1 [file ijms-24-15034-s001.zip › supplementary materials/Figure S3.jpg]

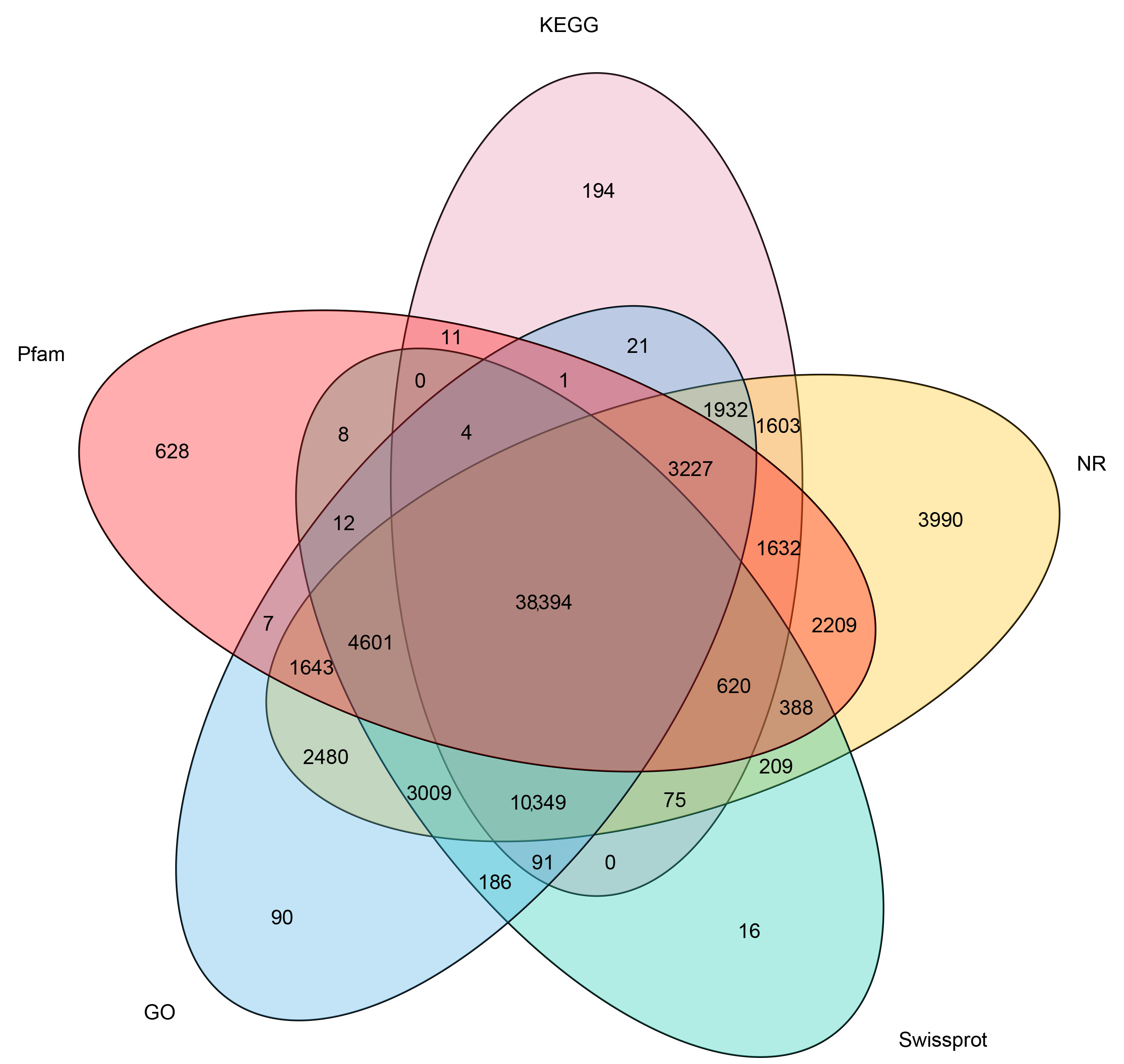

Supplement: Supplementary file 1 [file ijms-24-15034-s001.zip › supplementary materials/Figure S4.jpg]

Module-Traits Relationships

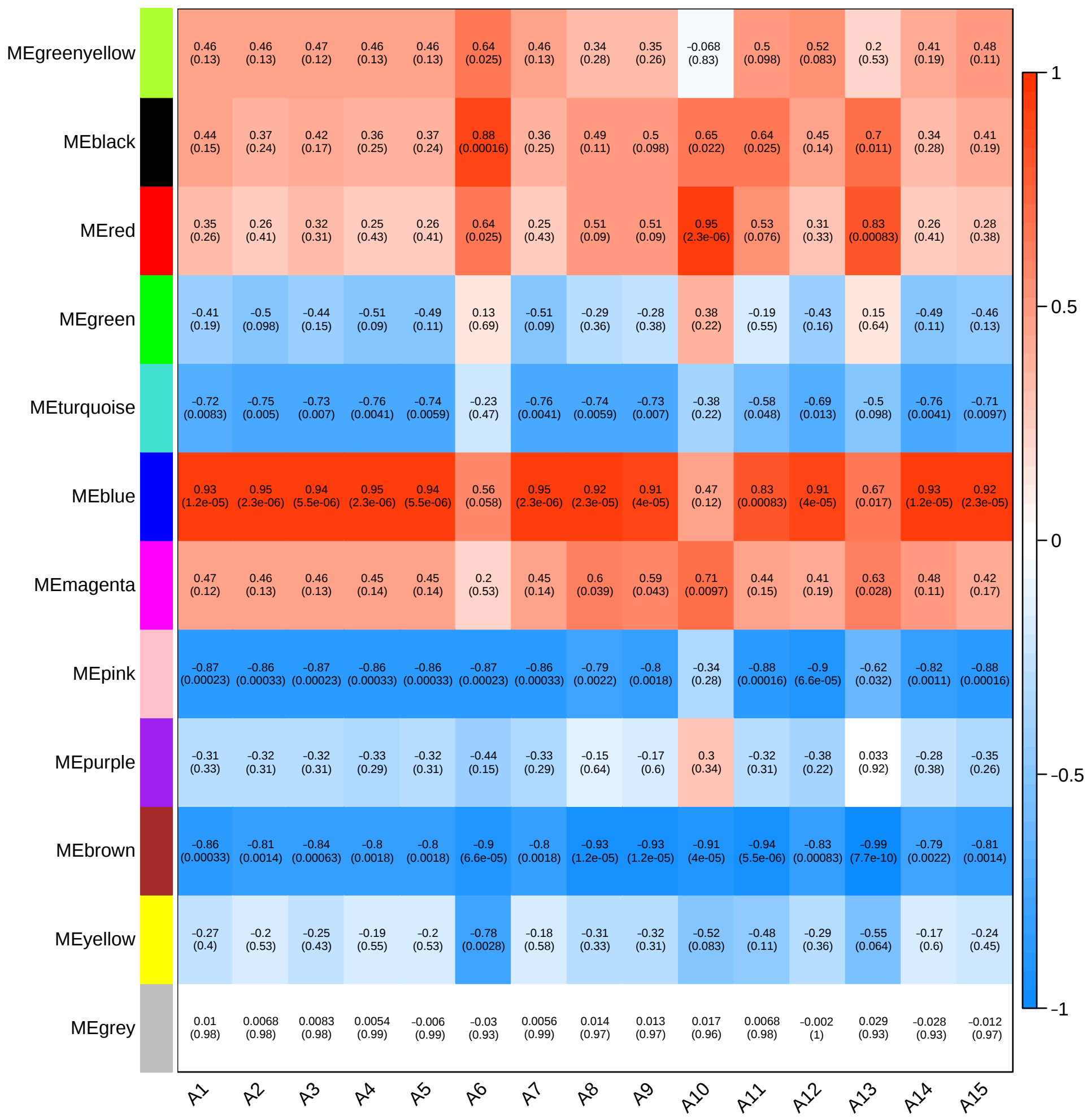

Supplement: Supplementary file 1 [file ijms-24-15034-s001.zip › supplementary materials/Figure S5.pdf]

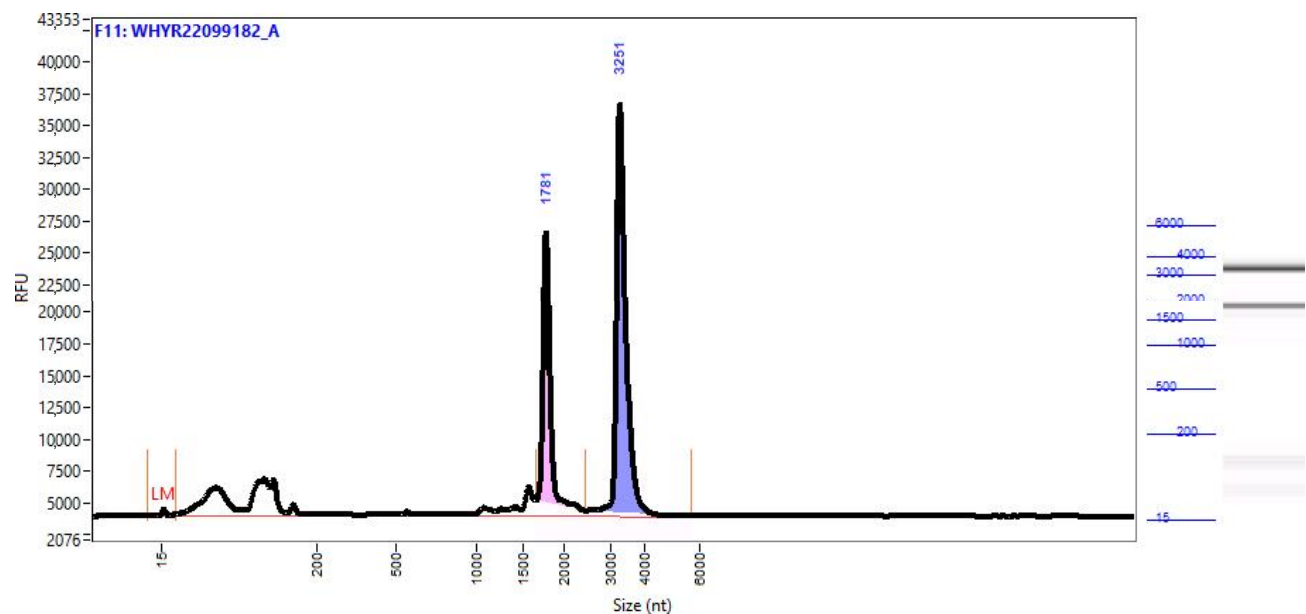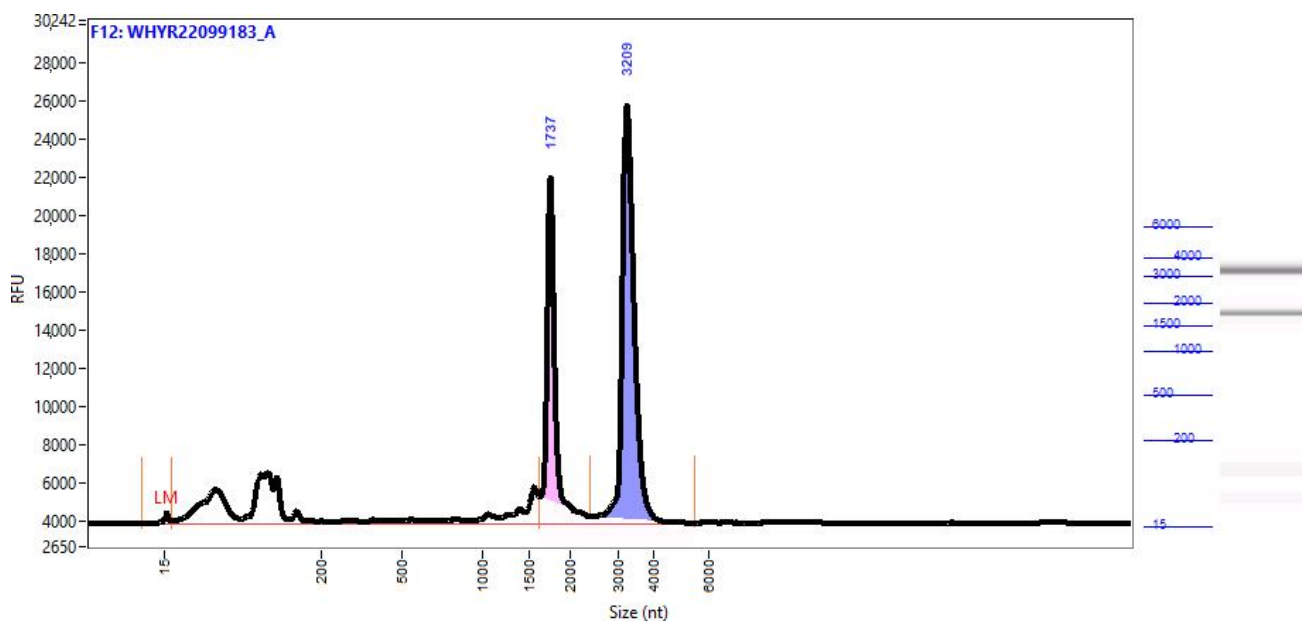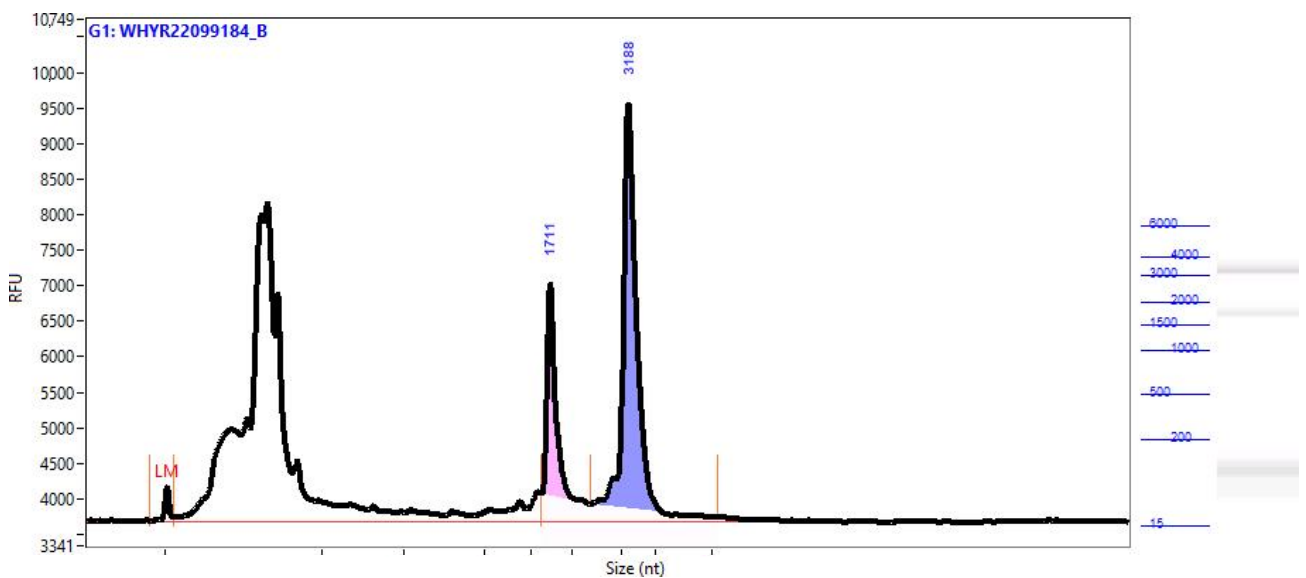

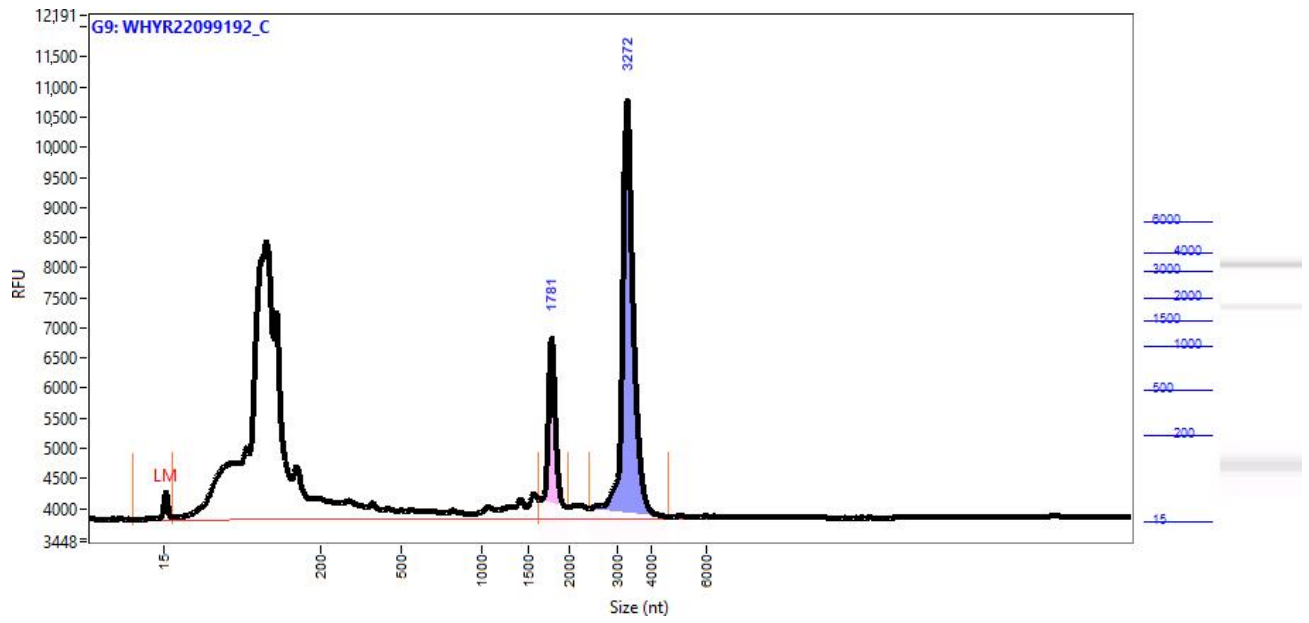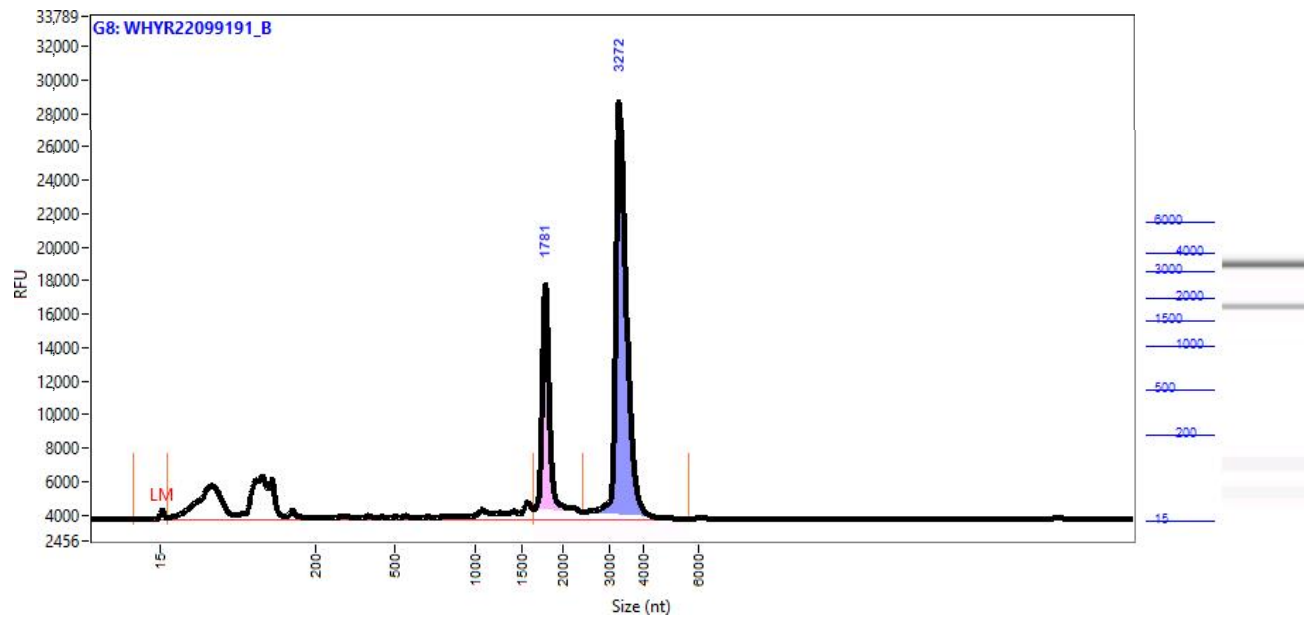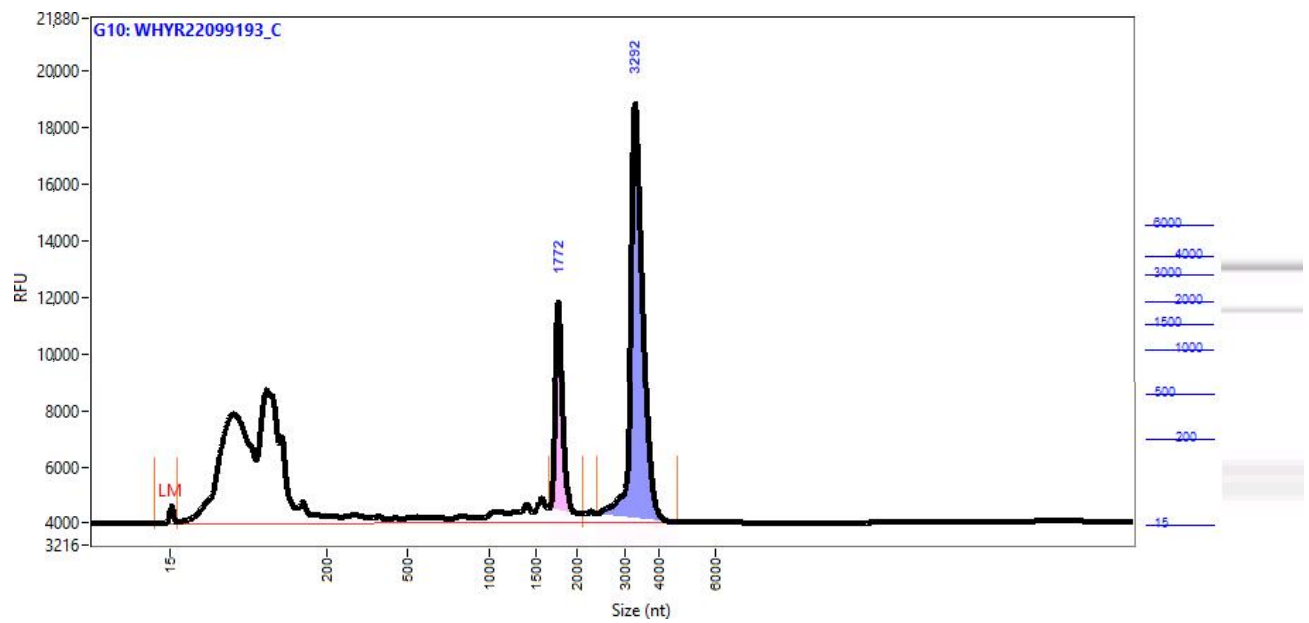

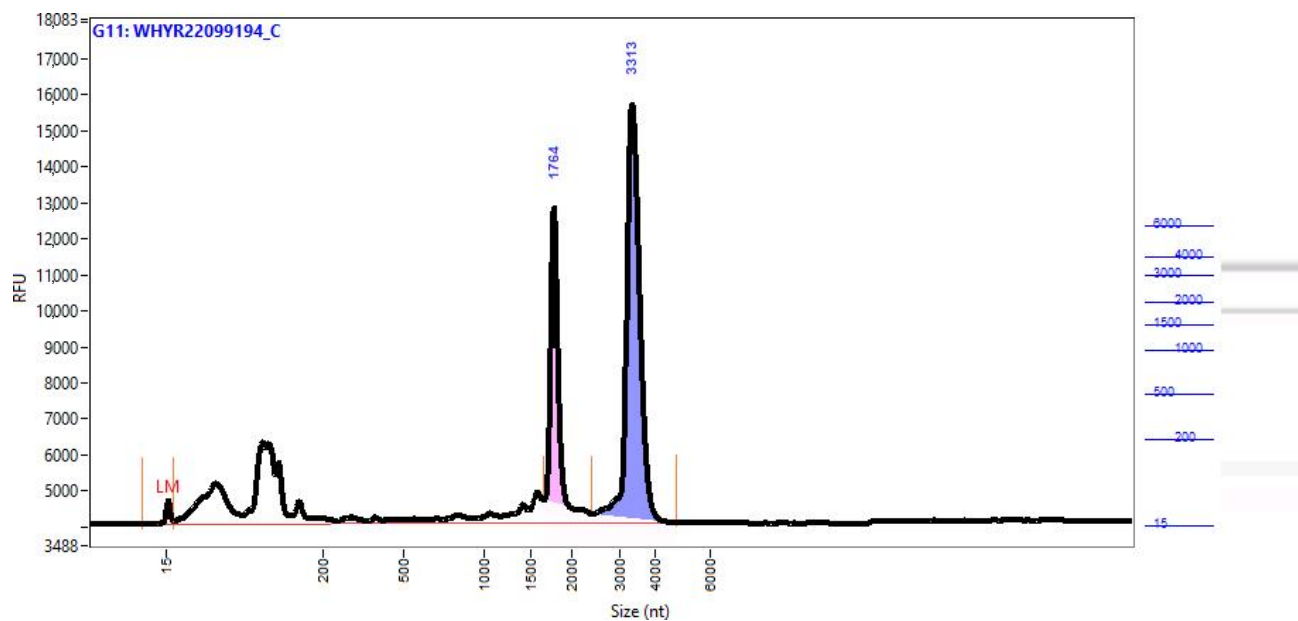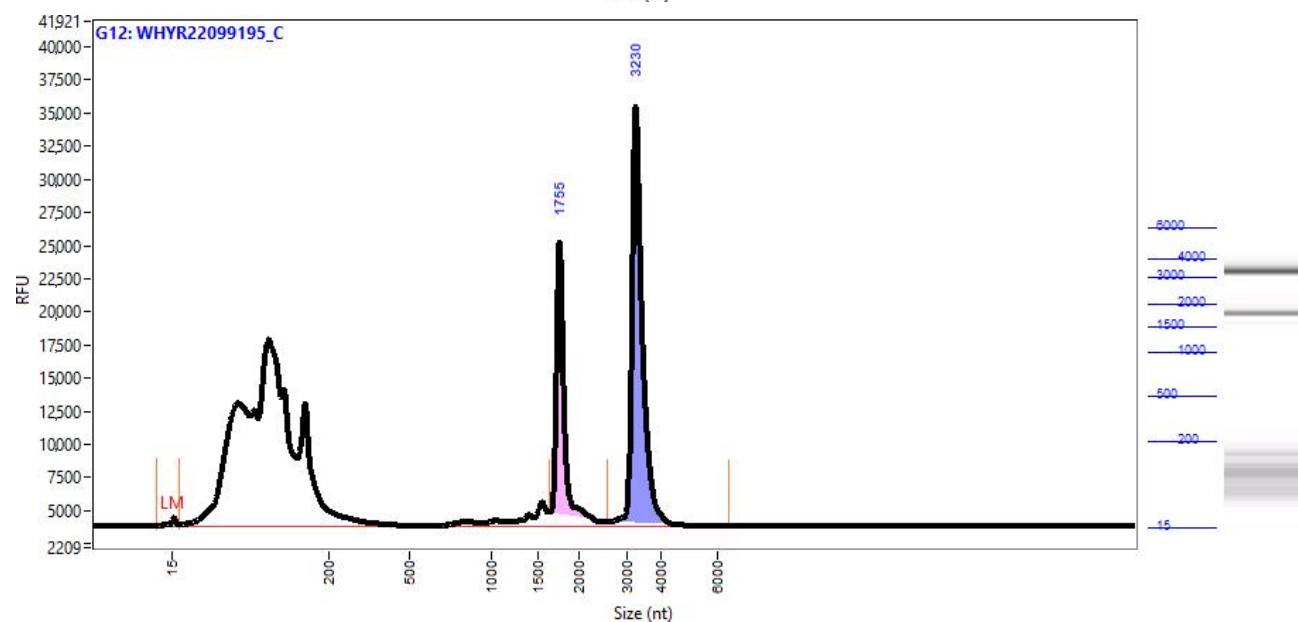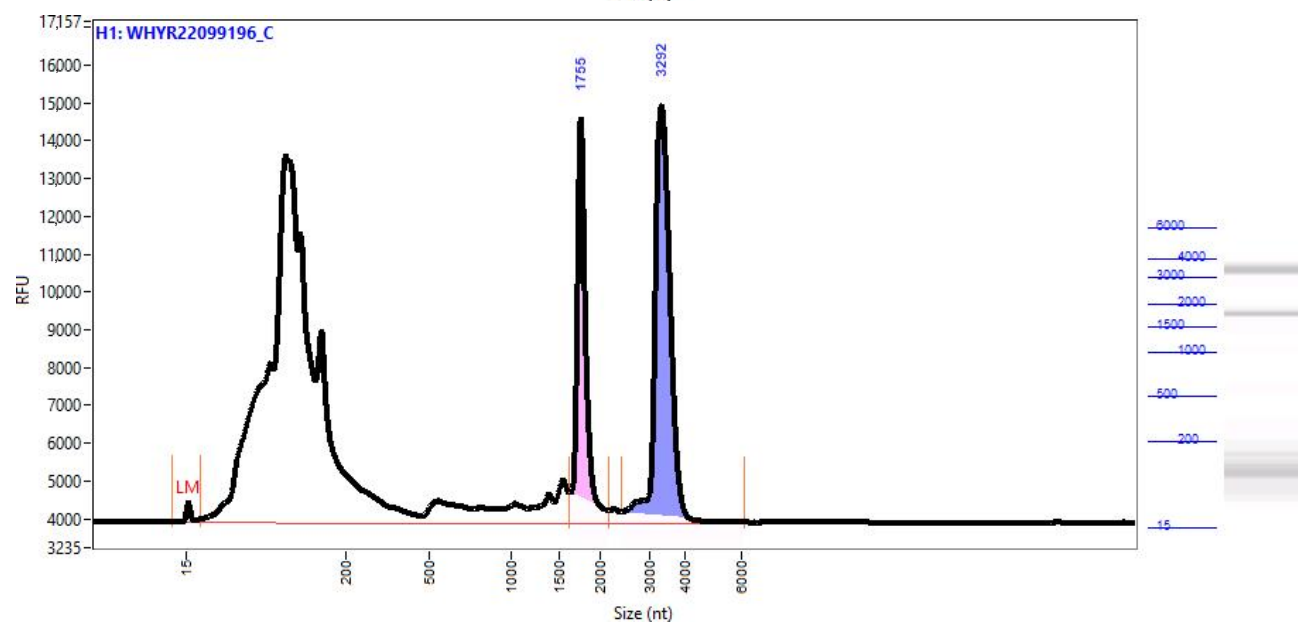

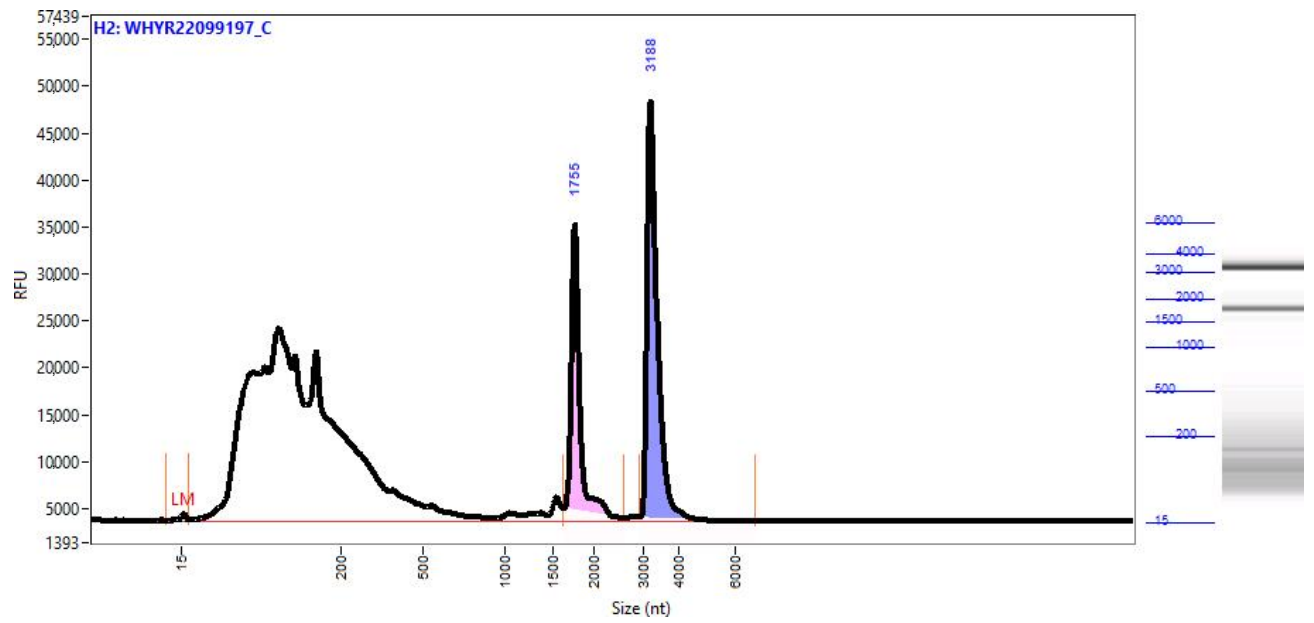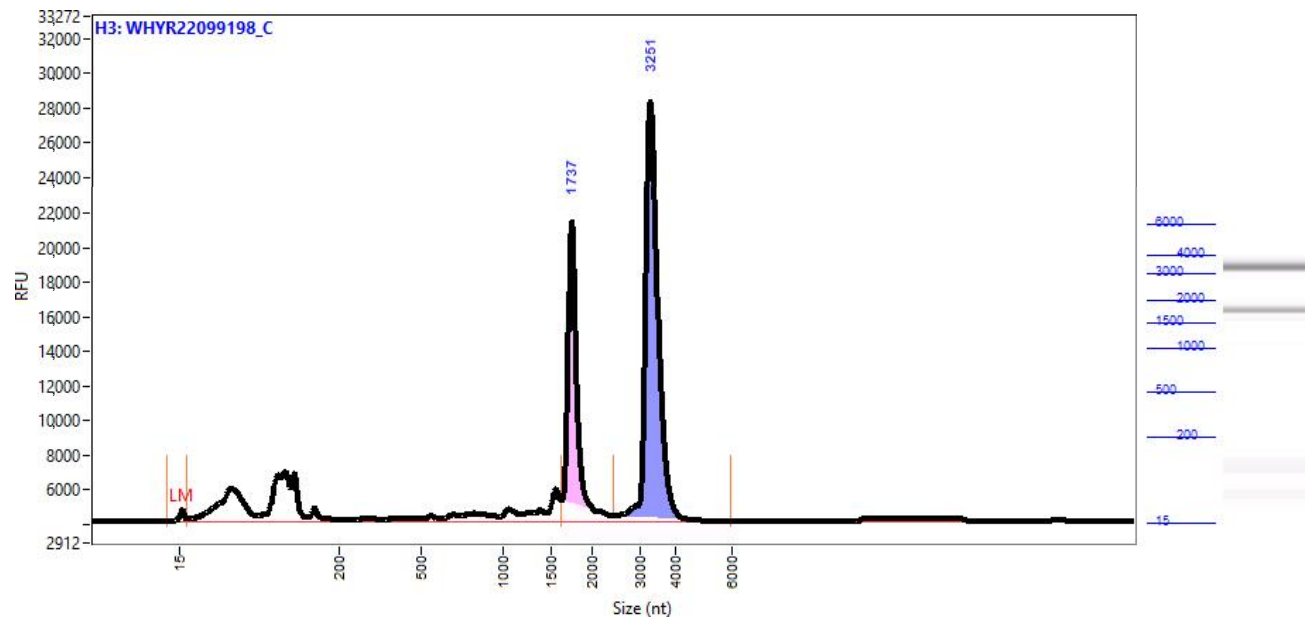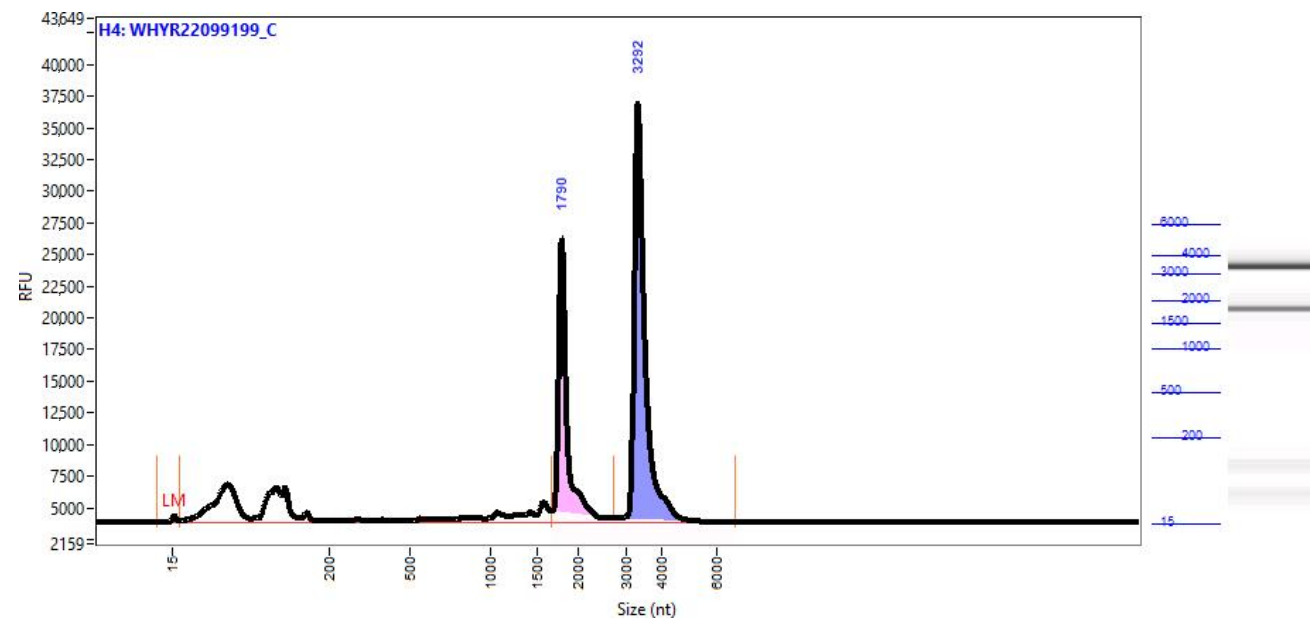

Supplement: Supplementary file 1 [file ijms-24-15034-s001.zip › supplementary materials/Figure S6.pdf]

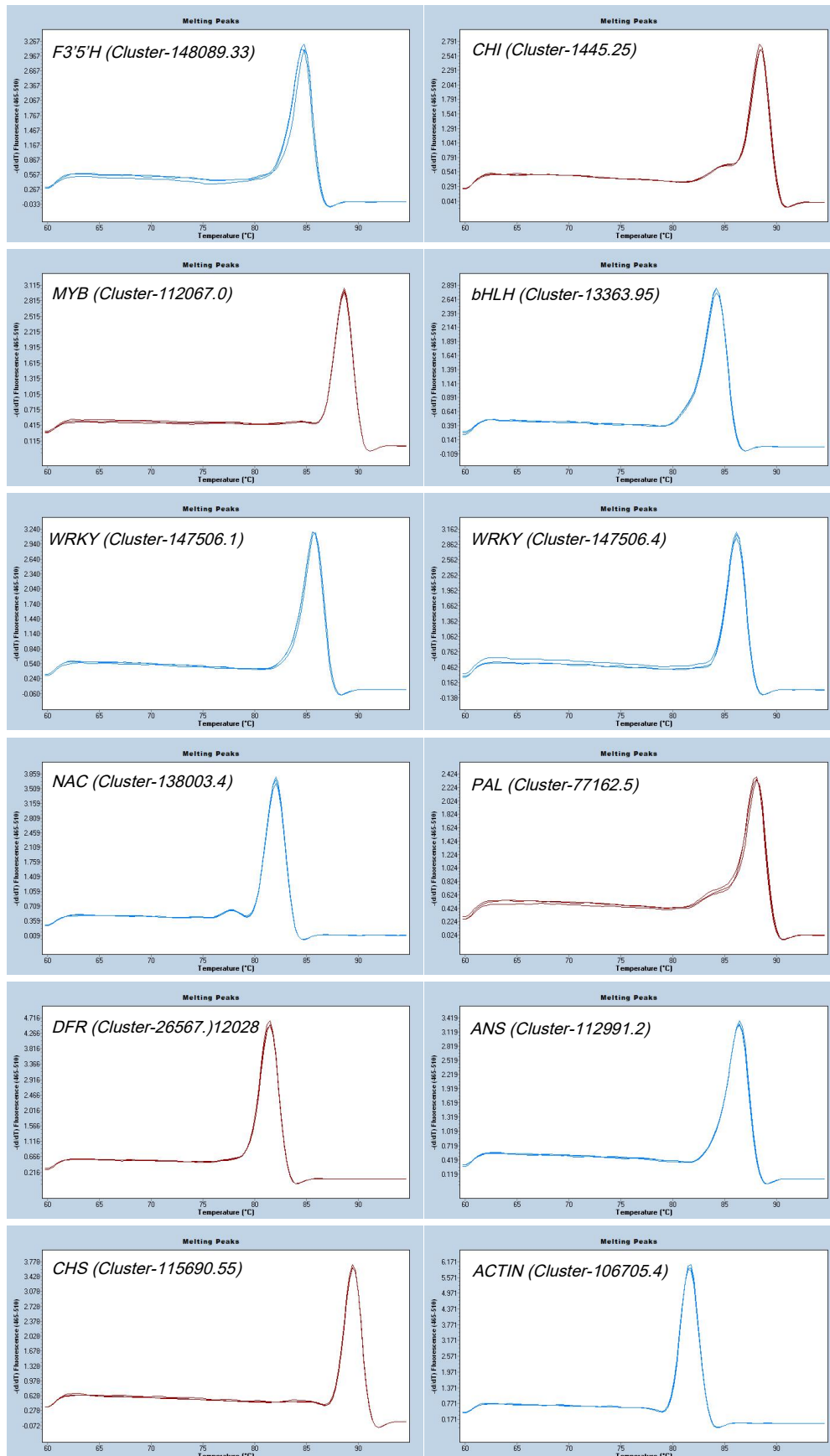

Supplement: Supplementary file 1 [file ijms-24-15034-s001.zip › supplementary materials/Figure S7.pdf]
